# Publisher Correction: Implementation of single-qubit measurement-based t-designs using IBM processors

**DOI:** 10.1038/s41598-022-10211-1

**Published:** 2022-04-11

**Authors:** Conrad Strydom, Mark Tame

**Affiliations:** Department of Physics, Stellenbosch University, Matieland, 7602 South Africa

Correction to: *Scientific Reports* 10.1038/s41598-022-08632-z, published online 23 March 2022

In the original version of this Article, Conrad Strydom was omitted as a corresponding author. Correspondence and requests for materials should also be addressed to conradstryd@gmail.com.

The original Article has been corrected.

