# Supplementary material for: Implementation of single-qubit measurement-based t-designs using IBM processors
Source: Sci Rep. 2022 Mar 23;12:5014. doi: 10.1038/s41598-022-08632-z (PMC8943145; doi:10.1038/s41598-022-08632-z)
Supplement: Supplementary file 1 — Supplementary Information. [file 41598_2022_8632_MOESM1_ESM.pdf]

Supplementary information for
Implementation of single-qubit measurement-based t-designs using IBM processors

Conrad Strydom* and Mark Tame
Department of Physics, Stellenbosch University, Matieland 7602, South Africa

I. QUBITS USED FOR THE 3-DESIGN

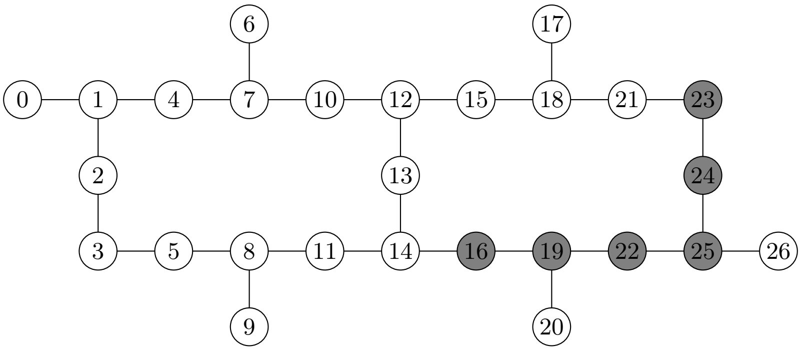

FIG. 1: Qubit topology of the *ibmq_ toronto* quantum processor. The connecting lines between qubits indicate the qubit pairs for which the CX gate is supported at the hardware level. The qubits used for the exact 3-design implementation are shaded gray.

The exact measurement-based 3-design was implemented on 6 physical qubits of the *ibmq_ toronto* quantum processor. This processor was chosen for its low error rates compared to other processors. The qubit topology of the *ibmq_ toronto* quantum processor is shown in Fig. 1. The qubits 1 to 6 of the 6-qubit linear cluster state in the 3-design implementation were mapped onto the physical qubits 16, 19, 22, 25, 24 and 23 of the *ibmq_ toronto* quantum processor, in such a way that the input state was prepared on qubit 16 and the output state was retrieved from qubit 23. These qubits were chosen as they form one of the few sets of 6 connected qubits (see Fig. 1) in which all the qubits generally have relatively low error rates. The relevant calibration information as obtained at the time of running the circuits for the exact 3-design implementation, is shown in Table I.

**Qubit**	T_1 **(μs)**	T_2 **(μs)**	$\sqrt{X}$ **Error**	**Readout Error**
16	123.42	135.48	0.000297	0.0154
19	114.98	123.10	0.000434	0.0116
22	110.50	148.90	0.000330	0.0191
25	125.41	114.64	0.000323	0.0108
24	121.49	155.26	0.000180	0.0093
23	98.92	40.59	0.000441	0.0553

**Qubit Pair**	CX **Error**
16–19	0.00758
19–22	0.01024
22–25	0.01053
25–24	0.01099
24–23	0.00892

TABLE I: Calibration information for the *ibmq_ toronto* quantum processor as obtained at the time of running the circuits for the exact 3-design. The single-qubit calibration information for the relevant qubits is shown on the left. T_1 and T_2 are the relaxation and dephasing times respectively of the qubits. The CX error rates for relevant qubit pairs are shown on the right.

*Electronic address: `conradstryd@gmail.com`

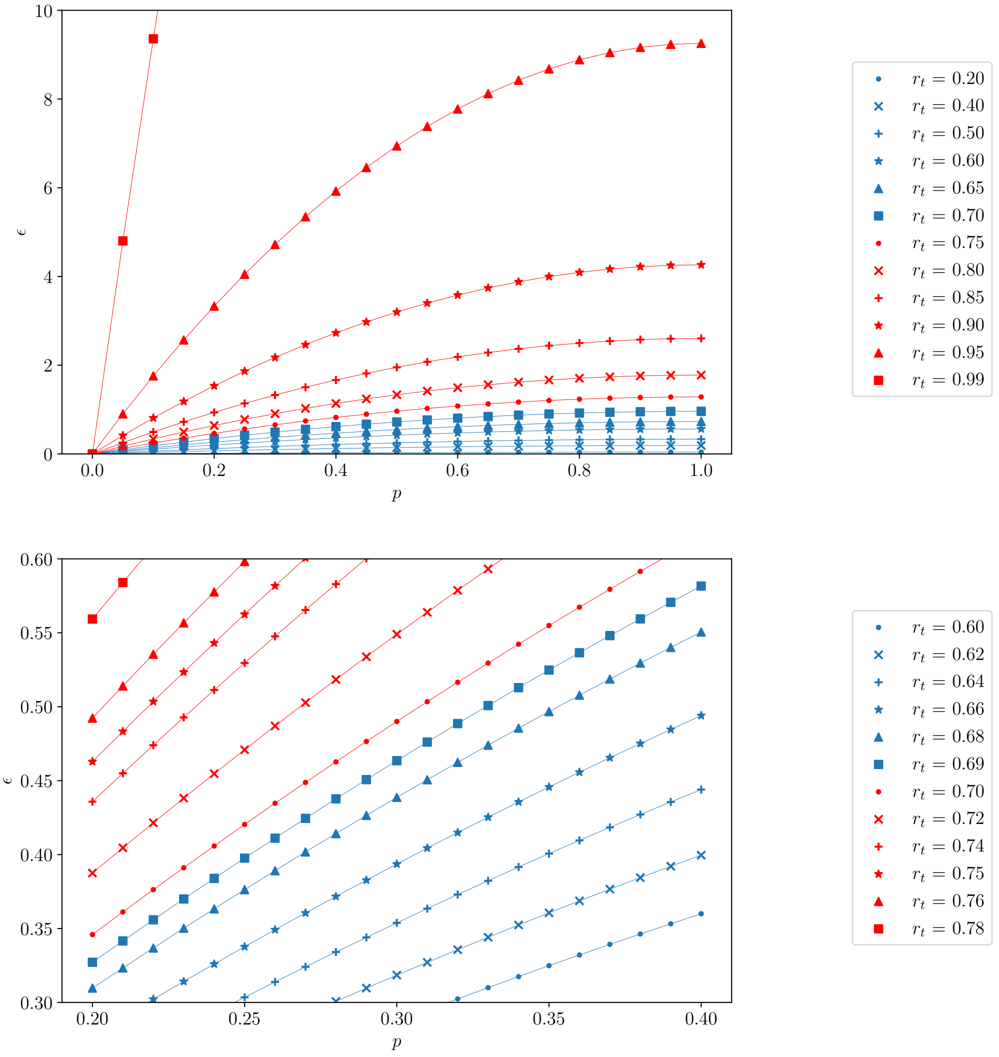

FIG. 2: ϵ versus p for the 2-design test with the middle term in inequality (9) of the main paper replaced by $\mathbb{E}_H^2(\rho'^{\otimes 2})$, for different truncation radii r_t. A plot of the full set of results is shown above and a plot focused on the region of interest is shown below.

II. DEPOLARISING NOISE

The action of the depolarising channel on a single-qubit state ρ is described by

$$\rho' = \frac{p}{2}I + (1-p)\rho, \tag{1}$$

that is, the state is replaced by the maximally mixed state with probability p. We investigated the effect of depolarising noise on an exact t-design's ability to accurately reproduce the moments of the uniform Haar ensemble. In particular, we carried out the test for an approximate t-design with the middle term in inequality (9) of the main paper replaced by $\mathbb{E}_H^t(\rho'^{\otimes t})$, the expectation of the uniform Haar ensemble computed from a state to which depolarising noise has been applied.

We managed to obtain test results analytically for the 1-design. Using the Pauli 1-design, $\{I, X, Y, Z\}$, we showed that $\mathbb{E}_H^1(\rho) = \frac{1}{2}I$ for all states ρ, which we used to show that $\epsilon = 0$ for all $p \in [0,1]$. Hence depolarising noise has no effect on a 1-design's ability to reproduce the first moment of the uniform Haar ensemble. Since $\mathbb{E}_H^2(\rho^{\otimes 2})$ and $\mathbb{E}_H^3(\rho^{\otimes 3})$ depend on the state ρ, analytic test results for the 2-design and 3-design are much harder to find. Using a sample of 1000 density matrices, obtained as described in the "Testing for a t-design" section of the main paper, and computing $\mathbb{E}_H^2(\rho^{\otimes 2})$ and $\mathbb{E}_H^3(\rho^{\otimes 3})$ for each density matrix ρ using the exact 3-design described in the "Measurement-based t-designs" section of the main paper, we obtained results numerically for the 2-design and the 3-design. Test

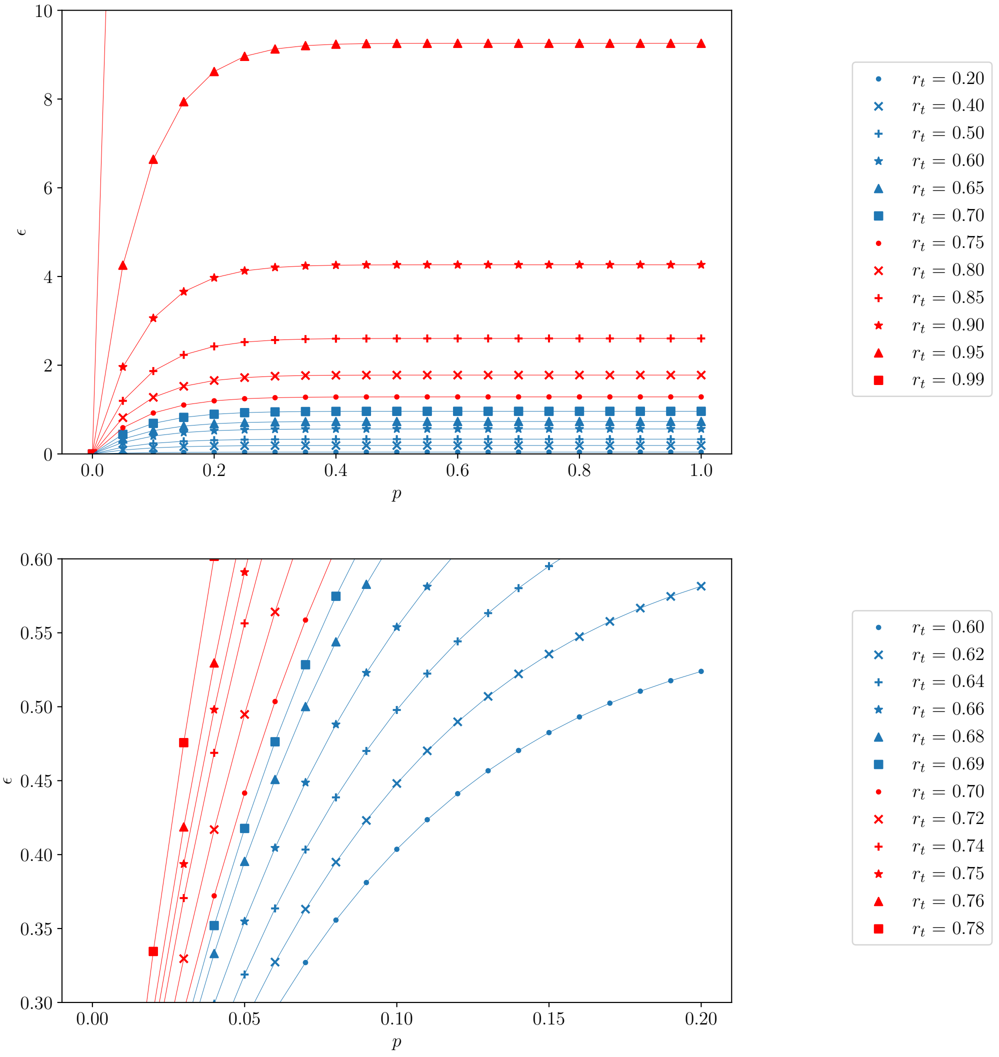

FIG. 3: ϵ versus p for the 2-design test with the middle term in inequality (9) of the main paper calculated by repeatedly applying depolarising noise to a state, in between the 5 individual unitary operations that are applied to the input state in the exact 3-design described in the "Measurement-based t-designs" section of the main paper, for different truncation radii r_t. A plot of the full set of results is shown above and a plot focused on the region of interest is shown below.

results obtained for the 2-design for different values of p and different truncation radii r_t are plotted in Fig. 2. For all truncation radii, ϵ increases linearly with p, up to about $p = 0.4$, after which the increase becomes more gradual. The values of ϵ obtained for states close to the surface of the Bloch sphere are very large, even for small p. This shows that the second moment of the uniform Haar ensemble is very sensitive to depolarising noise. Test results for the 3-design are identical to that of the 2-design, shown in Fig. 2. This suggests that the third moment of the uniform Haar ensemble is unaffected by depolarising noise.

To determine whether this depolarising noise model is a good noise model for the exact 3-design implementation on the *ibmq_toronto* quantum processor, we attempt to infer a consistent value for the parameter p from the test results in Table 3 of the main paper. Given r_t and ϵ obtained in a test for the 2-design or the 3-design, we simply read off the corresponding value of p from the plot in Fig. 2. Using the results for the 2-design test (without quantum readout error mitigation) we infer $p = 0.31$ and using the results for the 3-design test we infer $p = 0.36$. For the results with quantum readout error mitigation, we infer $p = 0.20$ using the 2-design test results and $p = 0.32$ using the 3-design test results. Since the values inferred from the 2-design test and the 3-design test are different in both cases, we conclude that this depolarising noise model is not the correct noise model for the 3-design implementation.

We now consider a depolarising noise model which more closely resembles the way in which depolarising noise occurs in t-designs generated using a measurement-based approach. In this model, the test for an approximate t-design is performed with the middle term in inequality (9) of the main paper calculated by repeatedly applying depolarising

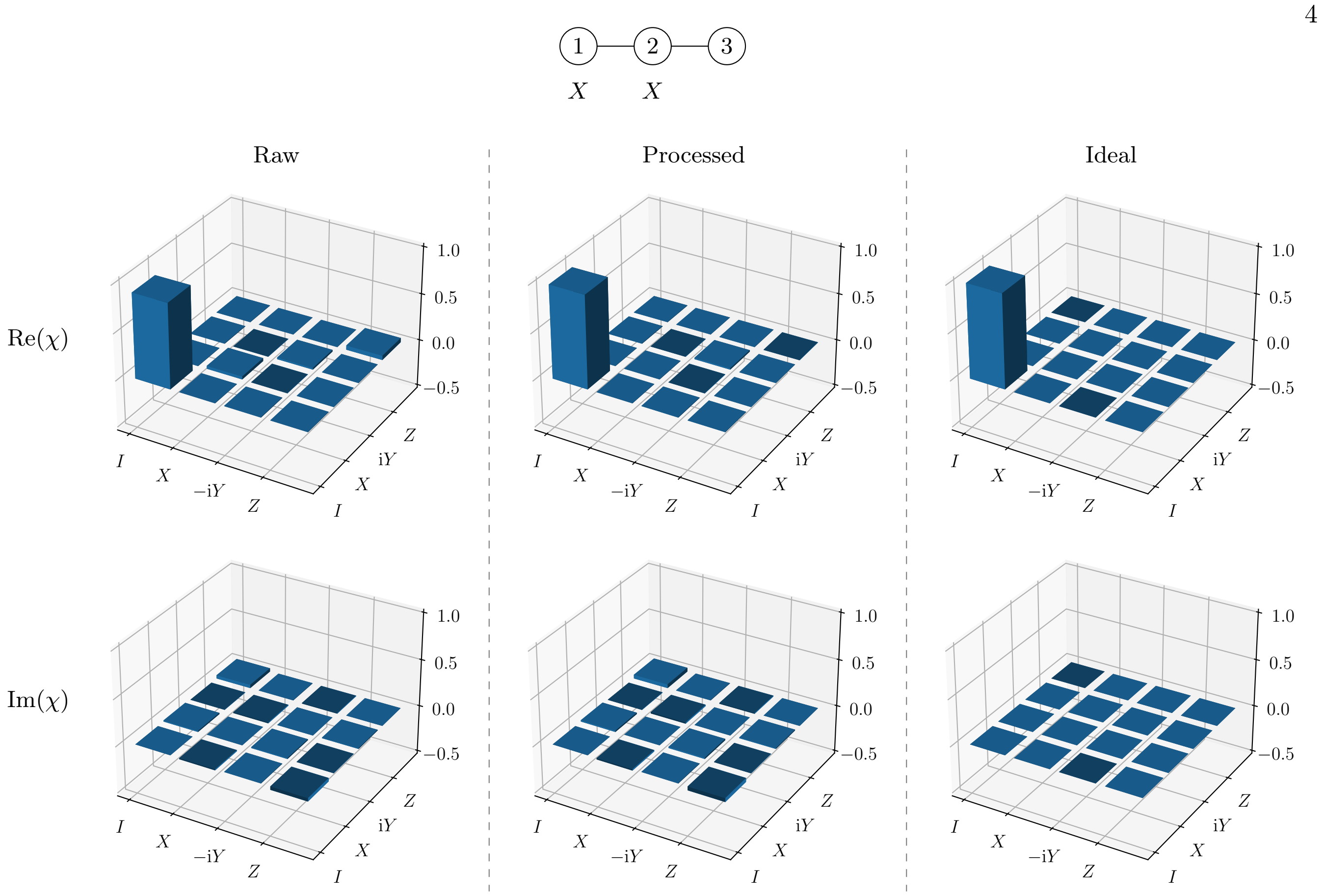

FIG. 4: Channel tomography results (average χ matrix) for the implementation of the identity operation with a 3-qubit cluster state on the *ibmq_toronto* quantum processor. The diagram at the top shows the entangled 3-qubit cluster state with the measurements performed on each qubit. The χ matrix obtained without quantum readout error mitigation is shown on the left, the χ matrix obtained with quantum readout error mitigation is shown in the middle and the ideal χ matrix is shown on the right. The real part of each matrix is shown above and the imaginary part of each matrix is shown below.

noise to a state, in between the 5 individual unitary operations that are applied to the input state in the exact 3-design described in the "Measurement-based t-designs" section of the main paper. Our analytic results for the 1-design carry over to this model. Test results obtained numerically for the 2-design are shown in Fig. 3. For this model, ϵ versus p starts to plateau at much smaller values of p. As a result of repeated applications of depolarising noise, the largest possible ϵ, for a given truncation radius, is reached for much smaller p. Results for the 3-design test are once again identical to that of the 2-design. Considering this model and using Fig. 3, we infer $p = 0.06$ using the results for the 2-design test (without quantum readout error mitigation) and $p = 0.07$ using the results for the 3-design test. For the results with quantum readout error mitigation, we infer $p = 0.04$ using the 2-design test results and $p = 0.06$ using the 3-design test results. The values of p inferred here from the two test results are close enough to be considered consistent in both cases. We therefore conclude that this is a very good noise model for the exact 3-design implementation on the *ibmq_toronto* quantum processor. From the values of p inferred, it is also clear that quantum readout error mitigation has reduced depolarising noise in the implementation.

III. IDENTITY IMPLEMENTATION

Measurement-based processing using n-qubit linear cluster states, as summarised in the "Measurement-based t-designs" section of the main paper, can be used to implement the identity operation for odd n. For measurements in the Pauli X-basis, that is in the direction $\phi = 0$, equation (1) of the main paper reduces to $U_m(0) = HZ^m$. Hence when all measurements on a n-qubit cluster state are performed in the Pauli X-basis, equation (2) of the main paper reduces to $U_{\boldsymbol{m}}(\mathbf{0}) = I$ for odd n when all measurement outcomes are 0. When some of the measurement outcomes are non-zero, the identity can still be implemented by performing the appropriate corrective (Pauli) operations on the output state [1]. For example, for the 3-qubit cluster state and the measurement outcome $\boldsymbol{m} = 10$, the operation

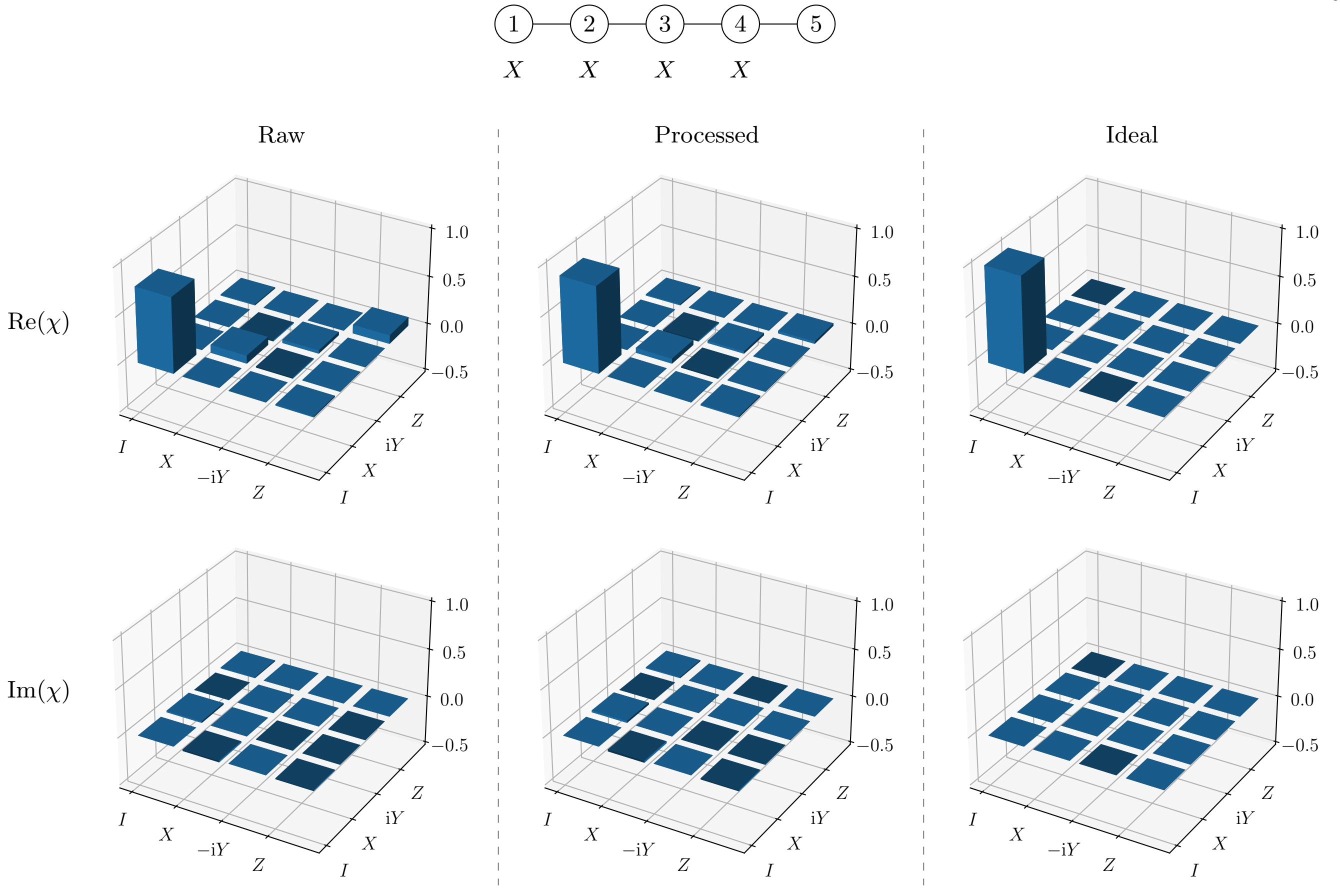

FIG. 5: Channel tomography results (average χ matrix) for the implementation of the identity operation with a 5-qubit cluster state on the *ibmq_toronto* quantum processor. The diagram at the top shows the entangled 5-qubit cluster state with the measurements performed on each qubit. The χ matrix obtained without quantum readout error mitigation is shown on the left, the χ matrix obtained with quantum readout error mitigation is shown in the middle and the ideal χ matrix is shown on the right. The real part of each matrix is shown above and the imaginary part of each matrix is shown below.

$HZH = X$ will be implemented and the identity can be implemented by applying the Pauli X operation to the output state. Measurement-based implementations of the identity operation can be used to determine the type of noise on a set of qubits. For depolarising noise, we expect non-zero real entries along the diagonals of χ matrices obtained by channel tomography.

We implemented the identity operation by performing single-qubit measurements on 3-qubit, 5-qubit and 7-qubit linear cluster states prepared on the same qubits of the *ibmq_toronto* quantum processor as was used for the exact 3-design implementation. Sec. V provides more detail on the qubits used. Generation of channel tomography results, combining of counts to reduce statistical noise and construction of calibration matrices for quantum readout error mitigation were done in much the same way as for the exact 3-design implementation on the *ibmq_toronto* quantum processor. The only significant difference is that we applied the appropriate corrective operations to the density matrices of the output states constructed by state tomography before using them to do channel tomography. The different χ matrices obtained by doing channel tomography for the different measurement outcomes were used to calculate an average χ matrix for each cluster state.

Channel tomography results (average χ matrices) obtained for the implementation of the identity operation with the 3-qubit, 5-qubit and 7-qubit linear cluster states are displayed in Figs. 4, 5 and 6 respectively. Non-zero real entries are clearly visible along the diagonals of constructed χ matrices, which confirms that depolarising noise was indeed the predominant type of noise for these qubits. Comparing the channel tomography results for the different cluster states, we see that as the length of the cluster used in the implementation increases, so does the depolarising noise in the implementation.

We now infer a value for the parameter p for the depolarising noise present in each implementation of the identity. To this end, we model depolarising noise in one of these measurement-based implementations as a channel in which depolarising noise is applied to the input state n times for an implementation with a n-qubit cluster state. For each implementation, we considered 10000 evenly spaced values of p in the range 0 to 1. For each p, we determined the

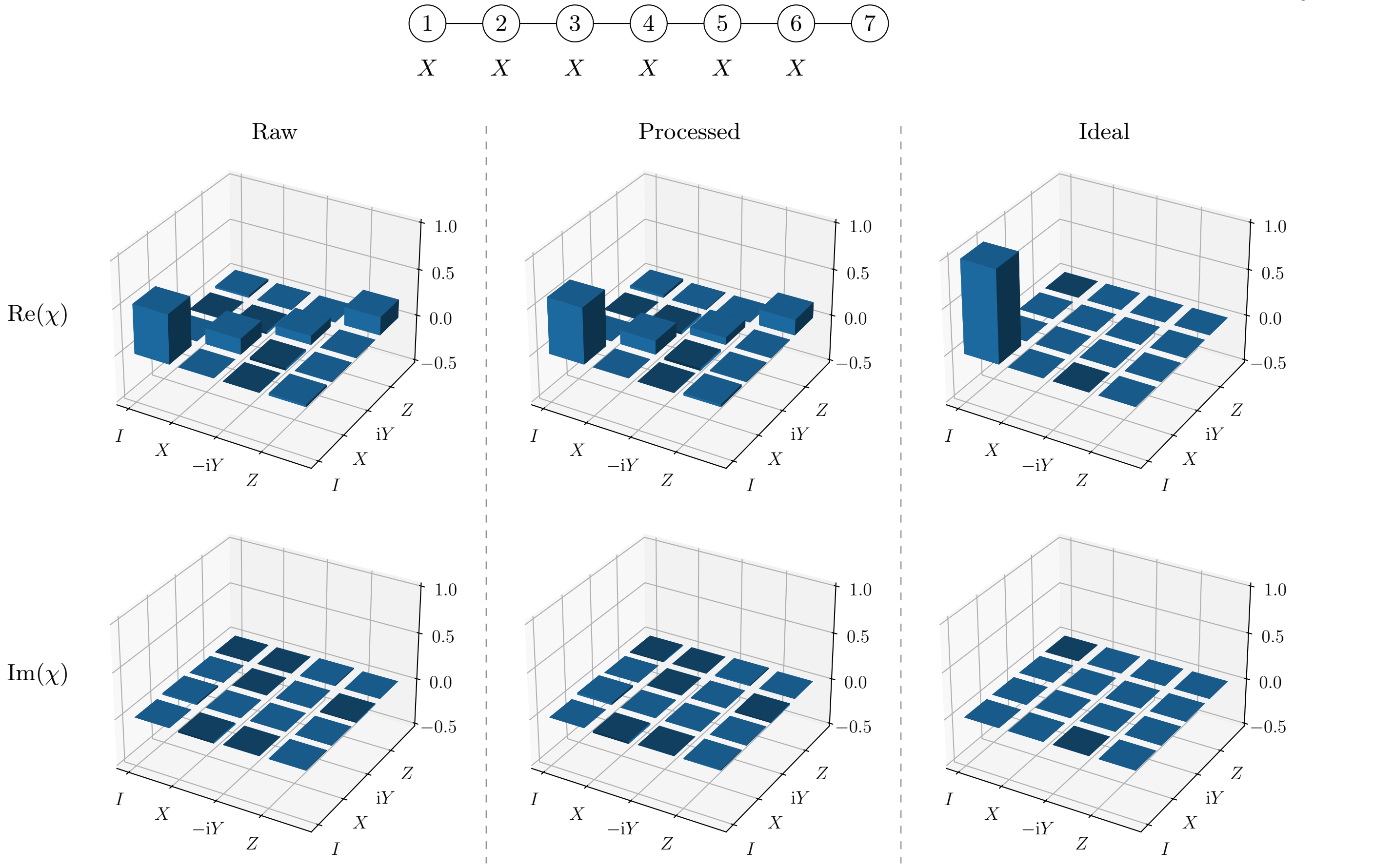

FIG. 6: Channel tomography results (average χ matrix) for the implementation of the identity operation with a 7-qubit cluster state on the *ibmq_toronto* quantum processor. The diagram at the top shows the entangled 7-qubit cluster state with the measurements performed on each qubit. The χ matrix obtained without quantum readout error mitigation is shown on the left, the χ matrix obtained with quantum readout error mitigation is shown in the middle and the ideal χ matrix is shown on the right. The real part of each matrix is shown above and the imaginary part of each matrix is shown below.

**Cluster State**	**p (Raw)**	**p (Processed)**
3-qubit	0.042	0.004
5-qubit	0.062	0.027
7-qubit	0.127	0.099

TABLE II: Values of p inferred for implementations of the identity with different cluster states on the *ibmq_toronto* quantum processor. 'Raw' shows the values of p without quantum readout error mitigation. 'Processed' shows the values of p with quantum readout error mitigation.

χ matrix for the corresponding model channel and calculated the channel fidelity for the implemented channel using the average χ matrix. Our inferred value of p for a given implementation, is the one which yields the channel fidelity which is closest to 1. The values of p inferred for the different measurement-based implementations of the identity operation are given in Table II. These values quantify the increase in depolarising noise resulting from increasing the length of the cluster state. The values of p are greatly reduced by applying quantum readout error mitigation, which suggests that classical measurement errors are responsible for a substantial amount of depolarising noise in the implementations. Finally, we note that the values of p inferred for the identity implementation with the 5-qubit cluster state agree very well with the values of p inferred for the exact 3-design implementation with a 6-qubit cluster state on the same set of qubits. This shows that our methods used to infer the values of p are consistent.

IV. QUBITS USED FOR THE 2-DESIGN

The approximate measurement-based 2-design was implemented on 5 physical qubits of the *ibmq_sydney* quantum processor. Its qubit topology is identical to that of the *ibmq_toronto* quantum processor shown in Fig. 1. The qubits

Qubit	T_1 (μs)	T_2 (μs)	$\sqrt{X}$ Error	Readout Error
13	154.36	162.92	0.000172	0.0097
14	94.78	208.81	0.000206	0.0295
16	97.54	121.99	0.001255	0.0198
19	102.87	89.78	0.000382	0.0271
22	114.71	170.64	0.000233	0.0441

Qubit Pair	CX Error
13–14	0.00576
14–16	0.00621
16–19	0.01155
19–22	0.01168

TABLE III: Calibration information for the *ibmq_ sydney* quantum processor as obtained at the time of running the circuits for the approximate 2-design. The single-qubit calibration information for the relevant qubits is shown on the left. T_1 and T_2 are the relaxation and dephasing times respectively of the qubits. The CX error rates for relevant qubit pairs are shown on the right.

Qubit	T_1 (μs)	T_2 (μs)	$\sqrt{X}$ Error	Readout Error
16	123.42	135.48	0.000297	0.0154
19	114.98	123.10	0.000434	0.0116
22	110.50	148.90	0.000330	0.0191
25	125.41	114.64	0.000323	0.0108
24	121.49	155.26	0.000180	0.0093
23	98.92	40.59	0.000441	0.0553
21	76.60	56.22	0.000508	0.0177

Qubit Pair	CX Error
16–19	0.00758
19–22	0.01024
22–25	0.01053
25–24	0.01099
24–23	0.00892
23–21	0.01652

TABLE IV: Calibration information for the *ibmq_ toronto* quantum processor as obtained at the time of running the circuits for the identity implementation. The single-qubit calibration information for the relevant qubits is shown on the left. T_1 and T_2 are the relaxation and dephasing times respectively of the qubits. The CX error rates for relevant qubit pairs are shown on the right.

1 to 5 of the 5-qubit linear cluster state in the 2-design implementation were mapped onto the physical qubits 13, 14, 16, 19 and 22 of the *ibmq_ sydney* quantum processor, in such a way that the input state was prepared on qubit 13 and the output state was retrieved from qubit 22. The relevant calibration information as obtained at the time of running the circuits for the approximate 2-design implementation is shown in Table III. These 5 connected qubits (see Fig. 1) were chosen as they have unusually low error rates (in particular CX error rates) — much lower than the error rates of any 5 connected qubits on the *ibmq_ toronto* quantum processor. This is why the *ibmq_ sydney* quantum processor was used for this investigation instead of the *ibmq_ toronto* quantum processor. The *ibmq_ sydney* quantum processor was not considered for the exact 3-design implementation, as it does not have any 6 connected qubits with lower error rates than the 6 connected qubits of *ibmq_ toronto* quantum processor. In particular, the error rates of qubit 25, which is connected to the qubits used for the approximate 2-design implementation, are typically large.

V. QUBITS USED FOR THE IDENTITY

The identity operation was implemented on the *ibmq_ toronto* quantum processor (see Fig. 1 for the qubit topology) by performing single-qubit measurements on cluster states of different lengths. Qubits 1 to 3 of the 3-qubit linear cluster state were mapped onto the physical qubits 16, 19 and 22, qubits 1 to 5 of the 5-qubit linear cluster state were mapped onto the physical qubits 16, 19, 22, 25 and 24 and qubits 1 to 7 of the 7-qubit linear cluster state were mapped onto the physical qubits 16, 19, 22, 25, 24, 23 and 21. Qubits were chosen in this way to ensure maximum possible overlap with the qubits used for the exact 3-design implementation. This allowed us to compare depolarising noise parameters inferred for the two implementations. The relevant calibration information as obtained at the time of running the circuits for the identity implementation is shown in Table IV.

[1] Nielsen, M. A. Cluster-state quantum computation. *Rep. Math. Phys.* **57**, 147–161 (2006).
